# Polydopamine nanoparticle-based colorimetric–photothermal dual-mode lateral flow immunoassay for thromboembolism diagnosis

**DOI:** 10.1039/d6ra04164g

**Published:** 2026-08-12

**Authors:** Jun Yin, Yu Qiu, Yao Nie, Jiansheng Ju, Yanmin Ju, Fei Wang

**Affiliations:** a Taixing Hospital of Traditional Chinese Medicine Taizhou Jiangsu 225400 China; b College of Pharmacy, China Pharmaceutical University Nanjing Jiangsu 211198 China juyanmin@cpu.edu.cn

## Abstract

Lateral flow immunoassay (LFIA) using gold nanoparticles (AuNPs) as signal labels is widely utilized for point-of-care diagnostics. However, it is typically limited by a single colorimetric readout and insufficient sensitivity. To address these limitations, polydopamine nanoparticles (PDANs) were introduced as alternative signal labels to enhance LFIA sensitivity. Owing to their high molar extinction coefficient and broadband absorption spanning the ultraviolet to near-infrared region, PDANs provide strong visual contrast and efficient photothermal conversion capability. Based on these advantages, a dual-mode colorimetric–photothermal LFIA platform was successfully constructed for the detection of thrombosis-related biomarkers, including thrombomodulin (TM) and α_2_-plasmin inhibitor–plasmin complex (PIC), in clinical serum samples. Under optimized conditions, the visual limits of detection (vLOD) for TM and PIC were 5 ng mL^−1^ and 50 ng mL^−1^, respectively. Subsequently, a convenient photothermal readout was introduced, and photothermal signal amplification further improved the sensitivity by approximately 10-fold for TM and 2.5-fold for PIC, achieving dual-mode detection. Clinical validation using 48 serum samples demonstrated good agreement with the clinical method. Overall, this PDANs-based LFIA platform enhances analytical sensitivity and reliability, providing a feasible strategy for the advancement of LFIAs.

## Abstract 2

Dual-mode colorimetric–photothermal LFIA enables sensitive, simple point-of-care testing.

## Graphical abstract

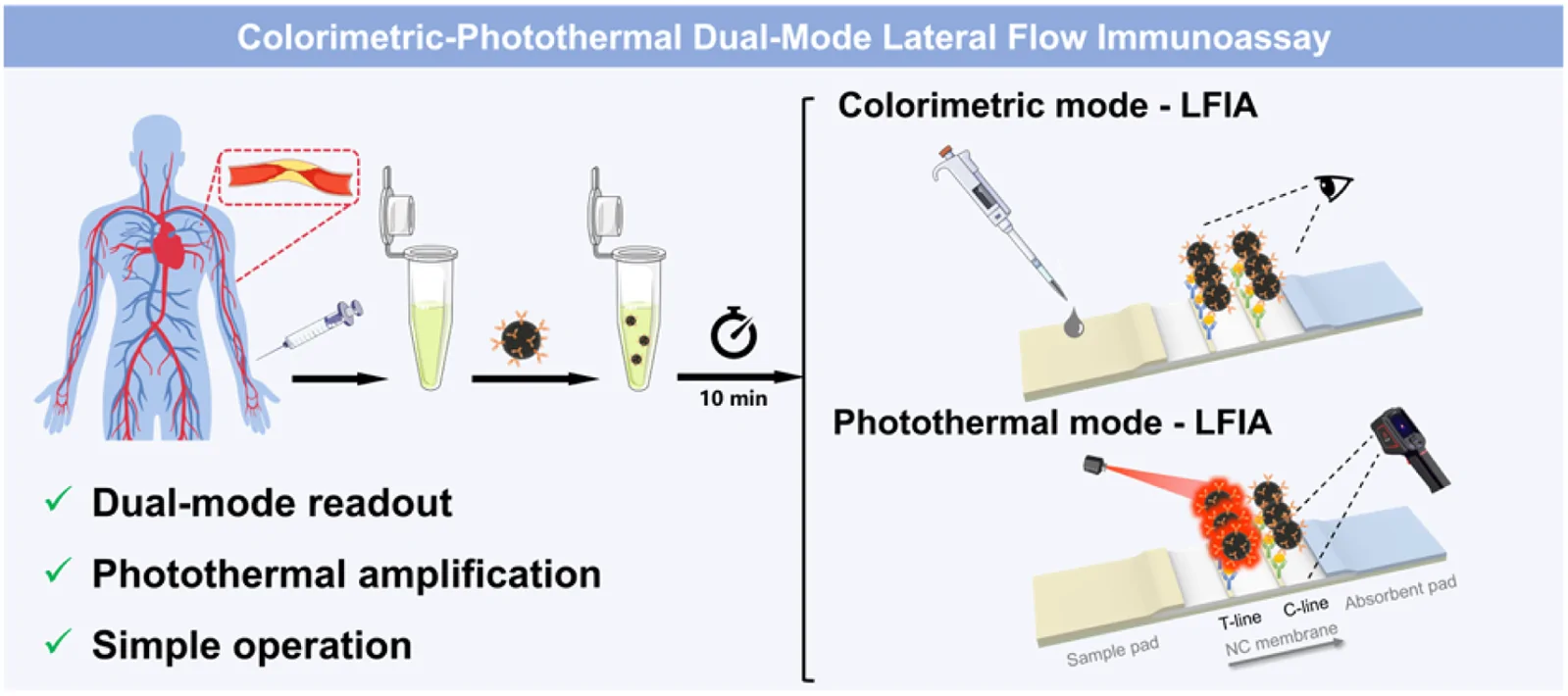

## 1 Introduction

The lateral flow immunoassay (LFIA) is a solid-phase membrane immunoassay that integrates antigen–antibody specific recognition with nanomaterial labelling strategies, enabling the qualitative or quantitative detection of target analytes in complex matrices.^1–3^ Owing to its low cost, ease of operation, and independence from sophisticated instrumentation, LFIA has been extensively applied in fields such as *in vitro* diagnostics, food safety, and environmental monitoring.^4–6^ At present, conventional LFIA predominantly employs gold nanoparticles (AuNPs) as signal labels because of their distinct plasmonic color, facile preparation, good biocompatibility, and suitability for naked-eye visual detection. However, the colorimetric signal intensity of AuNPs labels may be insufficient for low-abundance targets, thereby limiting detection sensitivity.^7,8^ Moreover, a single colorimetric readout inherently depends on variations in color intensity for result interpretation. In the low-concentration range, such visual signals are susceptible to interference from background coloration, illumination conditions, and subjective judgment, leading to compromised quantitative capability and reduced analytical reliability.^9–11^ To overcome these challenges, recent research has sought to enhance the analytical performance of LFIA by optimizing signal reporters and expanding detection modalities.

Replacing AuNPs with nanomaterials possessing high molar extinction coefficients has been widely recognized as an effective strategy to enhance colorimetric signal intensity and improve the sensitivity of LFIA. For example, black nanomaterials,^12,13^ carbon-based nanomaterials^14,15^ and plasmonic nanostructures^16^ have been employed as signal labels to improve the visual contrast and analytical sensitivity of test lines. However, this approach remains confined to a single colorimetric output mode. Under complex matrix conditions or at low analyte concentrations, the quantitative accuracy and resistance to interference are still limited. Therefore, dual-signal LFIA systems have attracted increasing attention, in which the conventional colorimetric readout is integrated with additional signal modes such as fluorescence,^17–19^ electrochemical,^20,21^ surface-enhanced Raman scattering,^22,23^ or photothermal signals.^24,25^ Although such dual-signal LFIA can improve detection sensitivity and analytical reliability to a certain extent, current systems often require specialized equipment and complex probes. These requirements limit their convenient application in home-based settings.

Polydopamine nanoparticles (PDANs) are melanin-like polymers formed *via* the spontaneous oxidative polymerization of dopamine under alkaline conditions.^26,27^ Owing to their high molar extinction coefficient, PDANs can significantly enhance the colorimetric contrast of test lines.^9,28–30^ Meanwhile, PDANs exhibit broadband absorption spanning from the ultraviolet to the near-infrared region, enabling efficient conversion of near-infrared light into heat. This excellent photothermal performance provides a practical material basis for constructing a dual-mode colorimetric–photothermal LFIA.^25,31^ Moreover, studies have demonstrated that the abundant quinone and phenolic hydroxyl groups on the surface of PDANs can form stable covalent bonds with antibodies, thereby simplifying the conjugation process and enhancing coupling efficiency.^32^ Combined with their favorable water dispersibility, chemical stability, and cost-effectiveness, PDANs exhibit significant potential as signal labels for constructing highly sensitive dual-mode LFIA platforms.

**Scheme 1:**
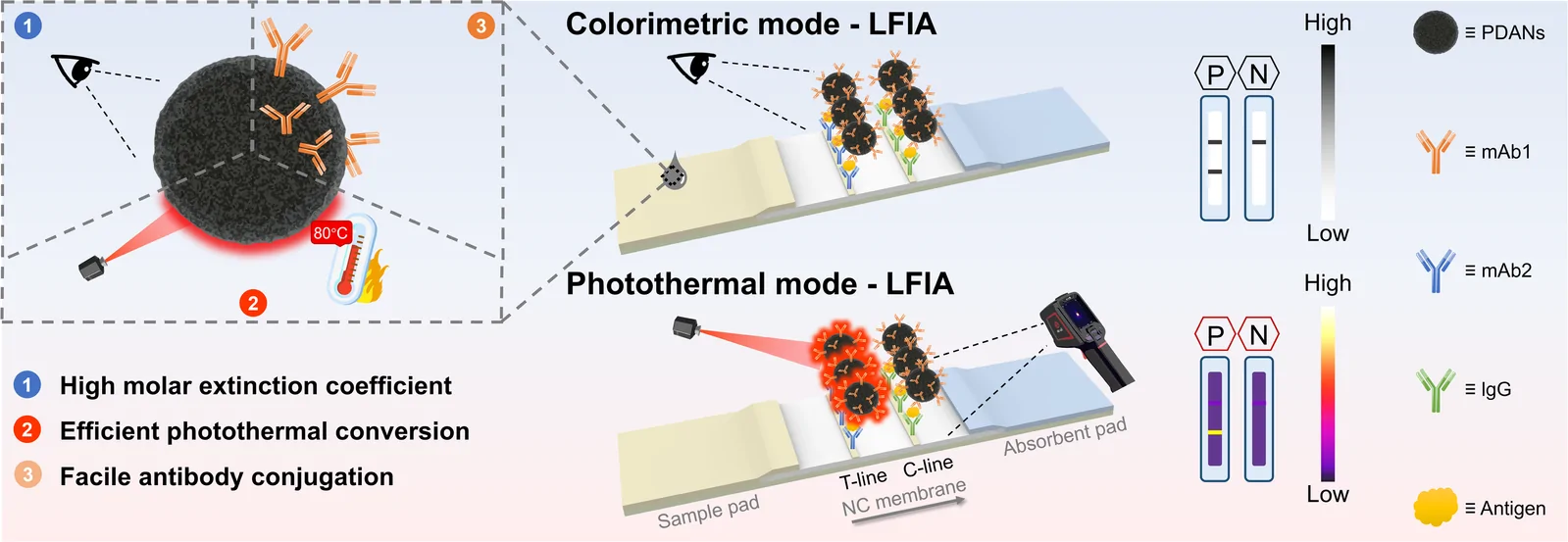
Schematic illustration of PDANs properties and the dual-mode colorimetric–photothermal detection process of the PDANs-based LFIA.

In this work, a PDANs-based LFIA platform was developed for the detection of thrombosis-related biomarkers, including thrombomodulin (TM) and α_2_-plasmin inhibitor–plasmin complex (PIC), to enhance early diagnostic performance and enable more accurate risk evaluation (Scheme 1).^33^ First, PDANs with an average particle size were successfully synthesized. Compared with AuNPs at the same concentration, PDANs exhibited more intense coloration and could be readily distinguished by the naked eye.^34^ Meanwhile, the abundant quinone groups on the surface of PDANs can form efficient and stable covalent bonds with antibodies by simple mixing, thereby significantly simplifying the conjugation process. Under optimal conditions, the visual limit of detection (vLOD) for TM and PIC was 5 ng mL^−1^ and 50 ng mL^−1^, respectively, using the PDANs-based LFIA platform. By utilizing the intrinsic photothermal properties of PDANs, the detection sensitivity was further enhanced by approximately 10-fold for TM and 2.5-fold for PIC, successfully achieving photothermal signal amplification and dual-mode colorimetric–photothermal readout. In addition, the detection results of 48 clinical serum samples showed good agreement with the reference clinical method, with an area under the receiver operating characteristic (ROC) value as high as 0.99. The PDANs-based LFIA platform preserves the operational simplicity of conventional LFIA while effectively improving detection sensitivity and reliability, offering a feasible strategy for advancing LFIA from single visual interpretation toward multimodal and quantitative analysis.

## 2 Materials and methods

### 2.1 Synthesis of PDANs

PDANs were synthesized *via* the oxidative self-polymerization of dopamine (DA). Firstly, NH_3_·H_2_O (70 µL), absolute alcohol (4 mL) and deionized water (9 mL) were mixed and stirred for 30 min. Then, 25 mg of DA was rapidly added in the mixture, which was subsequently stirred for 30 h, during which the color of the mixture gradually changed from colorless to pale yellow and to dark brown. The resulting solution was washed 3 times with deionized water by centrifugation (50 000×*g*, 30 min, 4 °C). The obtained PDANs were resuspended in deionized water and stored at 4 °C.

### 2.2 Preparation of antibody-conjugated PDANs

PDANs were conjugated with antibodies *via* strong quinone-amine interactions. First, PDANs (200 µL) were resuspended in 1 mL of PBS (0.01 M, pH 7.4), followed by the addition of anti-TM antibody (30 µg) or anti-PIC antibody (15 µg). The mixture was stirred for 90 min. Then 100 µL of 10% (w/v) BSA was added, the mixture was further stirred for 30 min to block the unreacted sites. The resulting solution was washed 3 times with PBS by centrifugation (10 947×*g*, 10 min, 4 °C). The obtained probes were resuspended in 200 µL of storage buffer (PBS containing 1% (w/v) BSA and 10% (w/v) sucrose) and stored at 4 °C.

### 2.3 Photothermal conversion performance of the PDANs

The photothermal performance of PDANs was evaluated to determine their feasibility as a second signal readout in LFIA. The temperature increase of PDANs was measured at different concentration (0–600 µg mL^−1^) and different powers (0–2.0 W). The photothermal conversion efficiency of PDANs was further evaluated under 808 nm laser irradiation (2.0 W) and a concentration of 600 µg mL^−1^. During irradiation, the temperature was recorded every 30 s using a thermal imaging camera. The photothermal conversion efficiency was determined from the heating and cooling curves based on the established heat transfer equation:^35^.1
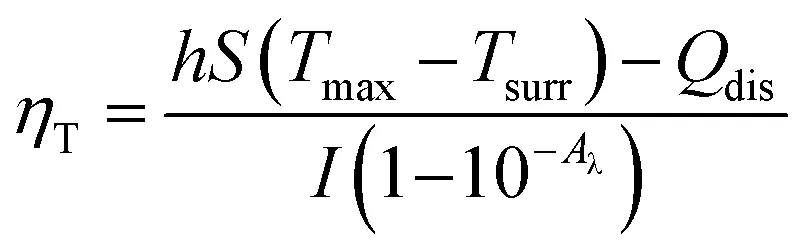
where *S* represents the specific surface area of the vessel, *h* represents the heat transfer coefficient, *T*_max_ and *T*_surr_ represent the maximum equilibrium temperature of the material and the ambient temperature, respectively. *A*_λ_ is the absorbance of the material at 808 nm. *I* represent the laser power, and *Q*_dis_ represents the change in heat of the water. The value of *hS* was determined by fitting the cooling curve according to eqn (1).2
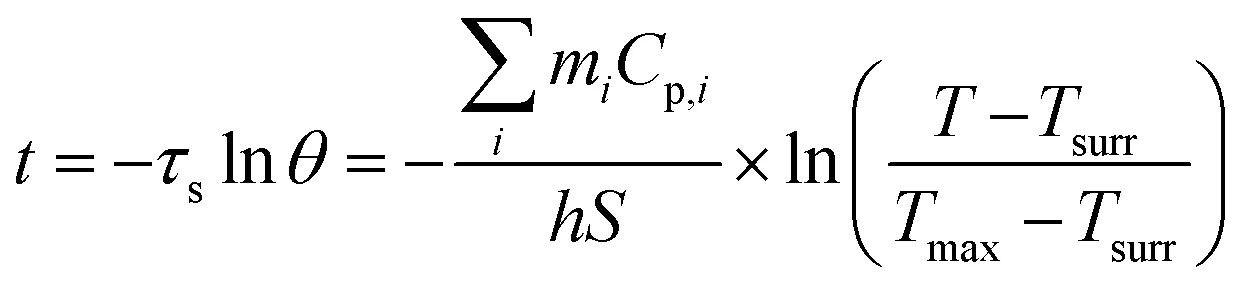
where *θ* represents the dimensionless thermal driving parameter and *τ*_s_ represents the time constant of the system, which can be obtained from the linear relationship between *t* and ln *θ*. The mass and specific heat capacity of water are denoted as *m*_*i*_ and *C*_p,*i*_, respectively. The corresponding parameter values used in the photothermal conversion efficiency calculation are summarized in Table S1.

### 2.4 Fabrication of LFIA test strips

The lateral flow test strip consisted of four functional components: the sample pad, NC membrane, PVC backing and the absorbent pad. Initially, the sample pad, NC membrane and the absorbent pad were assembled onto PVC background with an overlap of 1–2 mm between adjacent parts. Antibodies (1 mg mL^−1^) were dispensed onto the NC membrane at a rate of 1 µL cm^−1^: anti-TM antibody and goat anti-mouse Ig *G* were immobilized on the test (T) and control (C) lines, respectively, to fabricate the LFIA for TM detection, while anti-PIC antibody and goat anti-rabbit Ig *G* were immobilized on the corresponding T and C lines to fabricate the LFIA for PIC detection.

### 2.5 Detection of TM and PIC using LFIA strips

To evaluate the detection performance of the dual-mode LFIA strips, the prepared probes were incubated with a series of test solutions containing varying concentrations of TM or PIC for 10 min. After that, a blocking solution was added, and to the mixtures were applied onto the strips. During lateral flow, the probes specifically bound to their respective targets on the T line, while any unbound probes was captured on the C line. The test results were recorded using a camera under the same lighting conditions. The images were then imported into the “COLOR PICKER” software, and the RGB values were extracted on the T line of each strip (Fig. S1).

To further enhance the test signal, the strips were irradiated to NIR radiation (808 nm) at a power density of 2 W cm^−2^ for 1 min. The following thermal image was immediately acquired by a portable infrared camera. The temperature change (Δ*T*) on the T line was calculated according to the equation:^36^

3 Δ*T* = *T*_c_ − *T*_0_

where Δ*T* represents the temperature change between the test strip and the negative strip; *T*_c_ represents the temperature of the test strip at different analyte concentrations, and *T*_0_ represents the temperature of the negative strip.

### 2.6 Testing of clinical samples by PDANs-based LFIA

Clinical human serum samples (*n* = 48) including 18 positive samples and 30 negative samples, were obtained from the Taixing Chinese Medicine Hospital. The serum samples were diluted 5-fold with PBS prior to testing. The subsequent detection procedure followed the same protocol as that used for PIC detection. Each sample was tested in triplicate to ensure the reliability and reproducibility. All human serum samples were de-identified before analysis, and the study was approved by Taixing Hospital of Traditional Chinese Medicine (TXSZYY-2026-021).

## 3 Results and discussion

### 3.1 Synthesis and characterization of PDANs and PDNAs-mAb

**Fig. 1:**
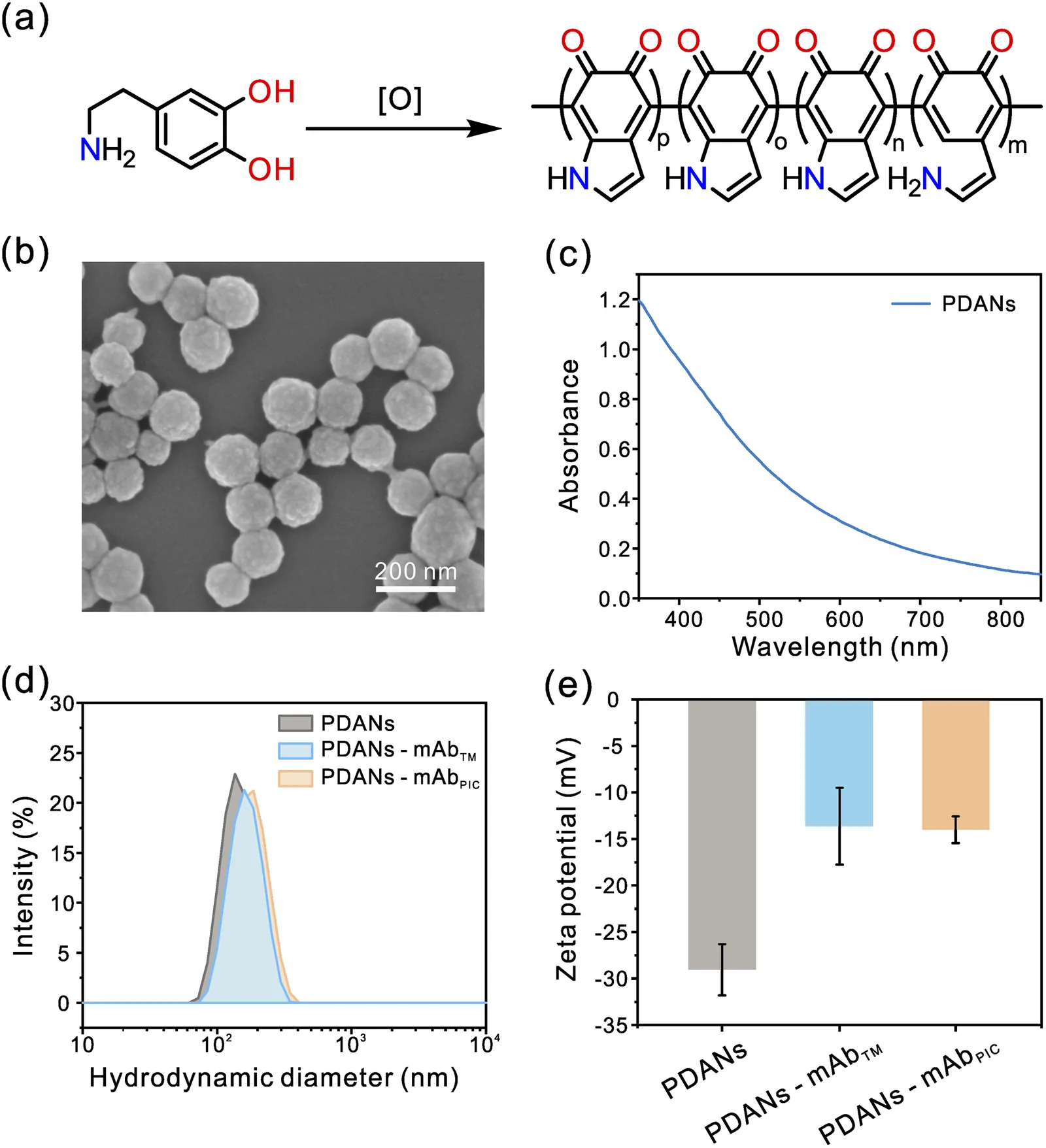
Synthesis and characterization of PDANs and PDANs-mAb probe. (a) Synthetic route of PDANs. (b) SEM image of PDANs. (c) The UV-vis absorption spectrum of PDANs. (d) Hydrodynamic diameter and (e) zeta potential of PDANs before and after antibody conjugation.

PDANs were formed by dopamine (DA) oxidation and self-polymerization under aerobic and alkaline conditions (Fig. 1a). PDANs with small diameters, which provided a balance color intensity and detection accuracy, were obtained by adding 70 µL NH_3_·H_2_O. meanwhile, scanning electron microscopy (SEM) images revealed that the PDANs exhibited a uniform spherical morphology with a diameter of around 150 nm (Fig. 1b) which was consistent with the hydrodynamic diameter of 154.5 nm measured by dynamic light scattering (DLS) (Fig. 1d). The UV-vis spectroscopy was conducted, and the obtained spectra were consistent with those reported in previous studies (Fig. 1c). In addition, the zeta potential of PDANs was −29.6 mV, suggesting that the PDANs possessed a strongly negatively charged surface (Fig. 1e). These results collectively confirmed the successful synthesis of PDANs. Three independently prepared PDANs batches showed consistent particle size distributions and photothermal performance, indicating good synthesis reproducibility and stable batch-to-batch properties (Fig. S2). Moreover, the stronger visual contrast of PDANs than conventional AuNPs at the same mass concentrations further supports their potential as colorimetric labels for LFIA (Fig. S3).

Due to the abundant quinone groups on the surface of PDANs, antibodies can readily form covalent bonds with PDANs through simple mixing.^37,38^ To verify the successful conjugation of antibodies, the hydrodynamic diameters of PDANs with TM mAb and PIC mAb were measured, and result showed that the diameter increased from 154.5 nm to 173.8 nm and 172.2 nm after conjugation, respectively (Fig. 1d). Moreover, after conjugation with TM and PIC mAb, the zeta potentials increased to −13.6 mV and −14.0 mV, respectively, indicating a less negative surface charge compared with pristine PDANs (Fig. 1e).

FTIR analysis was further performed to verify the antibody conjugation. The PDANs exhibited a broad band at 3475 cm^−1^, which can be attributed to the stretching vibration of phenolic O–H and N–H group. The characteristic band at around 1589 cm^−1^ was associated with the aromatic C=C stretching vibrations of the PDANs structure. After antibody conjugation, a new band appeared at 1629 cm^−1^, which can be attributed to amidel band^39^ (Fig. S4). The appearance of this amide-related band indicates the introduction of antibody proteins onto the PDANs surface. All these results indicated the successful preparation of the PDANs-mAb probes.

The storage stability of the PDANs-mAb probes was further evaluated. After storage for 0, 3, 7 and 10 days, the PDANs-TM mAb and PDANs-PIC mAb probes still maintained stable responses toward 100 ng mL^−1^ TM and PIC, respectively, indicating good storage stability (Fig. S5).

### 3.2 Evaluation of the photothermal performance of PDANs

**Fig. 2:**
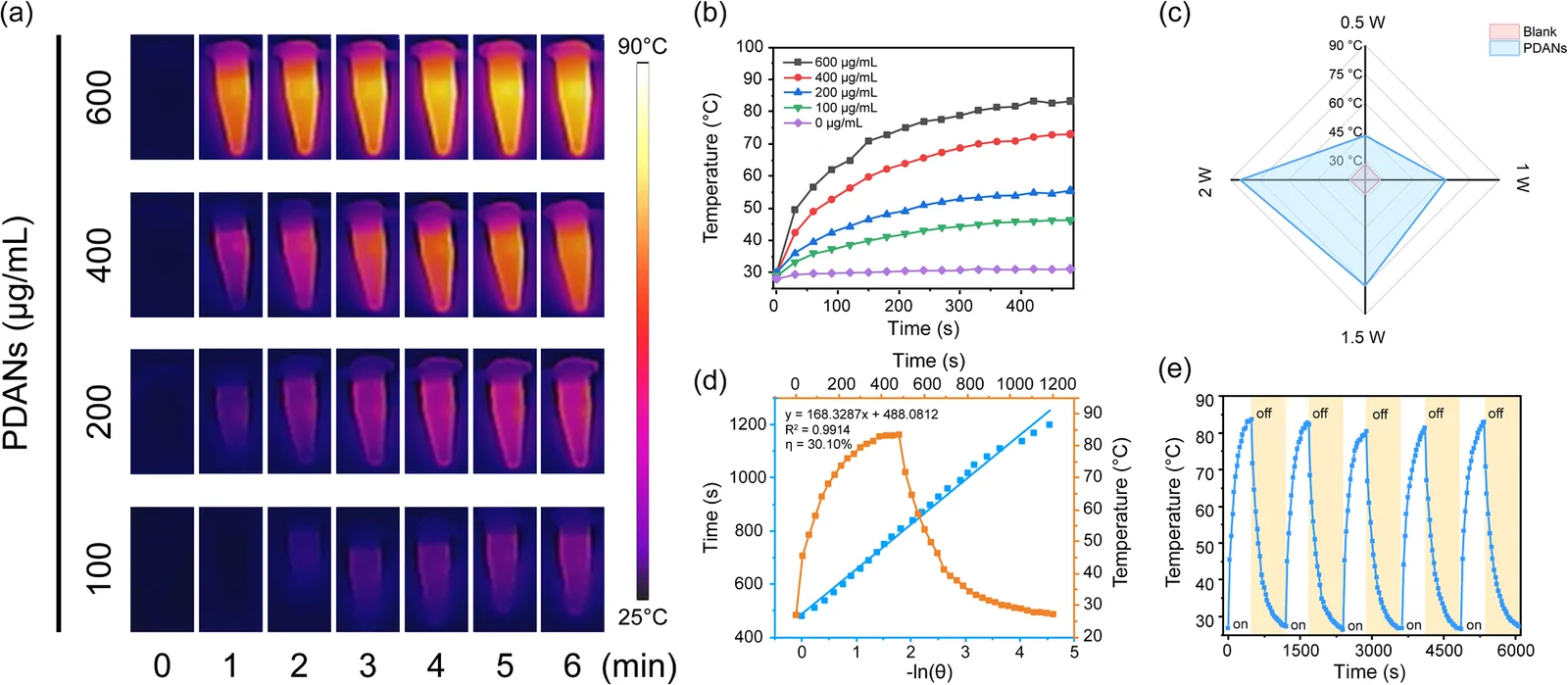
Photothermal properties of PDANs. (a) Thermal images and (b) photothermal heating curve of PDANs with different concentrations (808 nm, 2.0 W). (c) Photothermal heating characteristics of PDANs under different laser power irradiation (600 µg mL^−1^). (d) Heating and cooling curves of PDANs and plot of cooling time *versus* negative natural logarithm. (e) Heating and cooling curves of PDANs during five on/off cycles of 808 nm laser irradiation.

To more accurately evaluate the potential application of PDANs in dual-mode LFIA, the photothermal performance of PDANs was investigated. First, the UV-vis spectroscopy of PDNAs exhibited a broad absorption at 808 nm, indicating their potential as photothermal converters (Fig. 1c). Therefore, the temperature elevation of PDANs at different concentrations was evaluated under near-infrared laser irradiation (808 nm, 2 W, 6 min). As shown in Fig. 2a and b, the photothermal heating effect increased with increasing PDANs concentration, and the maximum temperature reached 83.4 °C at PDANs concentration of 600 µg mL^−1^. Under a power of 2 W, an obvious temperature increased were observed (Fig. 2c). Based on these results, the PDANs concentration of 600 µg mL^−1^ and the power of 2 W were selected for subsequent photothermal performance studies. Thereafter, the photothermal conversion efficiency (*η*) was determined from a single heating–cooling cycle experiment using PDANs, with pure water as the control. The temperature variation during the irradiation and cooling was recorded, and *η* was calculated according to eqn (1) and (2) to be 30.10% (Fig. 2d). Furthermore, the photothermal cycling behavior and stability of PDANs were investigated through consecutive laser on/off cycles. After 5 cycles, the maximum temperature of PDANs remained nearly unchanged, indicating excellent photothermal stability and reproducibility (Fig. 2e). These results demonstrate that PDANs exhibit excellent near-infrared responsiveness and high photothermal conversion efficiency. Highlighting their great potential as signal labels for dual-mode LFIA.

To evaluate the robustness of photothermal readout on LFIA strips, 100 ng mL^−1^ TM was used as a representative concentration, the effects of 808 nm laser power, irradiation time, and irradiation distance were investigated. Δ*T* increased with increasing 808 nm laser power, indicating that laser power is a critical parameter affecting the photothermal readout (Fig. S6a). Therefore, 0.5 W was used in all subsequent measurements to ensure readout stability and reproducibility. The temperature signal became stable after irradiation and showed no obvious increase after 60 s (Fig. S6b). Furthermore, Δ*T* showed only minor fluctuations within the tested distance range, suggesting that the photothermal readout was relatively stable under small variations in irradiation distance. These results indicate that the photothermal readout can provide stable signals on LFIA strips under standardized operating conditions.

### 3.3 Optimization of strip conditions

The PDANs-based LFIA was designed for the detection of TM and PIC. To achieve optimal detection performance, key parameters of the LFIA strip were systematically optimized, including antibody loading, probe volume, sample volume and immunoreaction time. For accurate signal quantification, the T-line intensities were extracted using the “Color Picker” software.

**Fig. 3:**
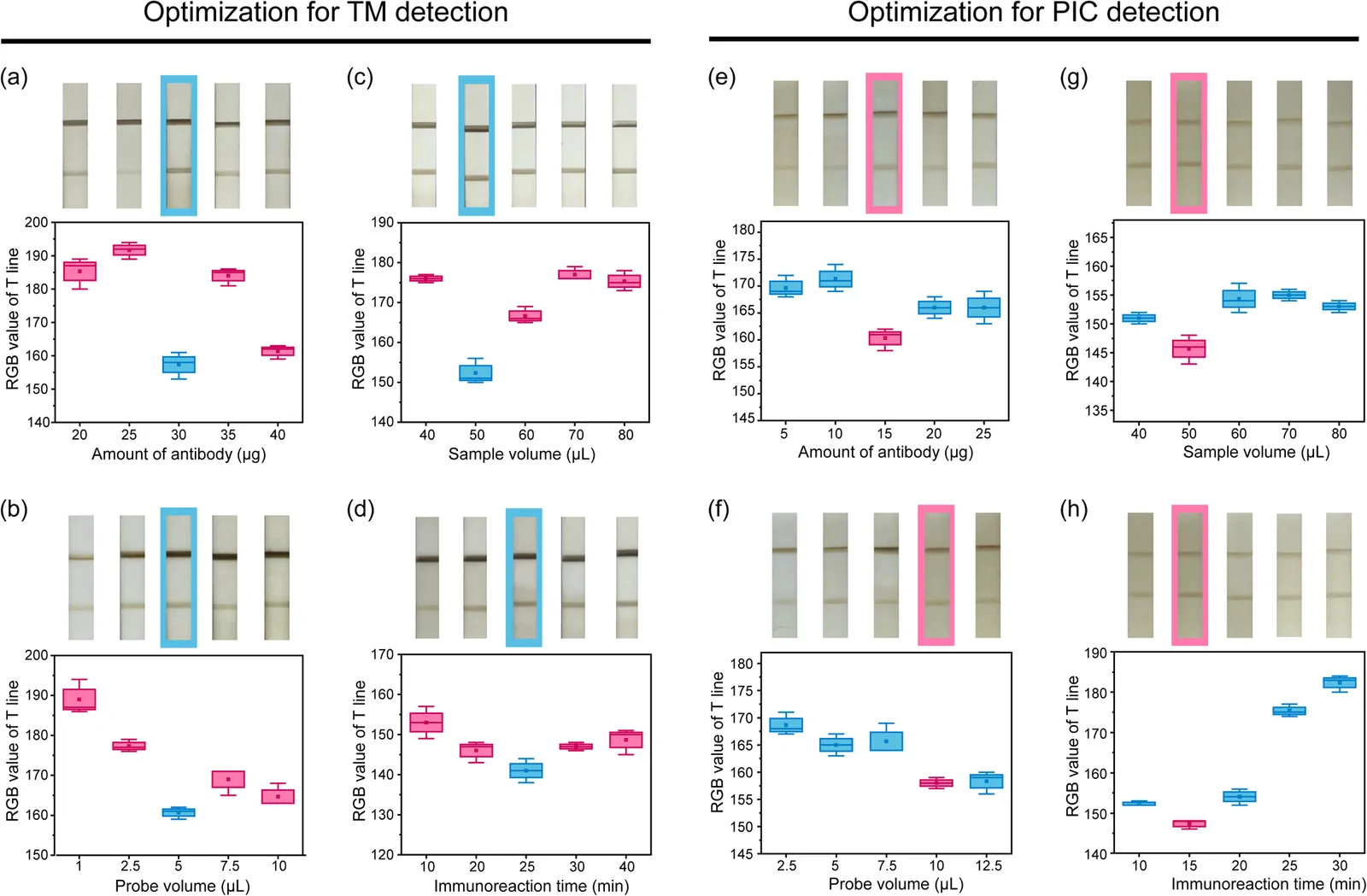
Optimization of the experimental parameters. Detection results of TM and PIC under varying (a and e) antibody amount, (b and f) probe volume, (c and g) sample volume, and (d and h) incubation time between sample and probe.

First, the PDANs-LFIA for TM detection was optimized. Different amounts of antibody (20, 25, 30, 35 and 40 µg) were evaluated to improve the detection sensitivity. The T-line intensity increased with increasing antibody loading, and reached the lowest RGB value at 30 µg of antibody, which was selected as the optimal antibody loading (Fig. 3a). The probe volume is a critical parameter in the LFIA platform, as excessive probe can increase background intensity and reduce T-line clarity. Therefore, a series of probe volumes (1, 2.5, 5, 7.5 and 10 µL) were investigated. When probe volume was 5 µL, an optimal balance between T-line and background signal was achieved (Fig. 3b). Similarly, the sample volume was optimal in the probe incubation step. As shown in Fig. 3c, the T-line exhibited the darkest band and the lowest RGB value when sample volume was 50 µL. Finally, the effect of immunoreaction time on T-line intensity was evaluated. The T-line intensity increased over time and reached a peak at 25 min, which was chosen as the optimal immunoreaction time for subsequent studies (Fig. 3d).

Meanwhile, the PDANs-based LFIA for PIC detection was similarly optimized for the same parameters. As shown in Fig. 3e–h, the platform exhibited optimal performance with 15 µg of anti-PIC antibody, a probe volume of 10 µL, a sample volume of 50 µL, and an immunoreaction time of 15 min, as indicated by the darkest T-line and the lowest RGB value. These optimized parameters were used in subsequent experiments to ensure high sensitivity and reproducibility of the dual-signal LFIA for TM and PIC detection.

### 3.4 Analytical performance of PDANs-based LFIA

The thrombotic biomarkers TM and PIC were detected using the PDANs-based LFIA platform. Under the optimized conditions, the analytical performance was systematically evaluated including qualitative and quantitative analyses.

**Fig. 4:**
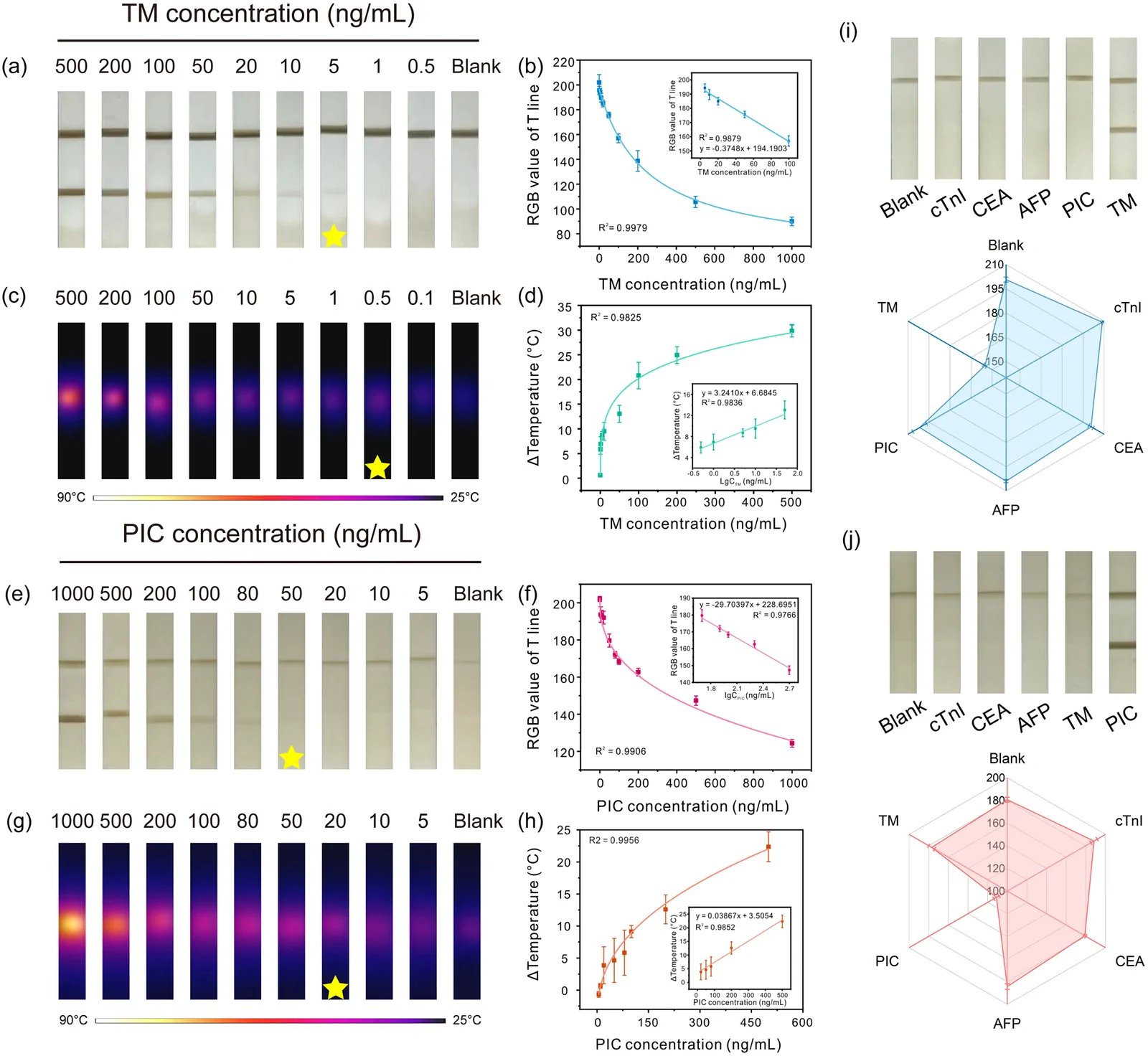
Analytical performance and specificity of the PDANs-based LFIA for TM and PIC detection. (a) Colorimetric assay images and (b) corresponding calibration curves for TM. (c) Photothermal assay images and (d) corresponding calibration curves for TM. (e) Colorimetric assay images and (f) corresponding calibration curves for PIC. (g) Photothermal assay images and (h) corresponding calibration curves for PIC. (i and j) Specificity evaluation of the PDANs-based LFIA for TM and PIC.

First, a series of standard TM (500, 200, 100, 50, 20, 10, 5, 1 and 0.5 ng mL^−1^) were analyzed using the PDANs-based LFIA. As shown in Fig. 4a, the T-line intensity gradually decreased with decreasing TM concentration, and the minimum detectable TM concentration was 5 ng mL^−1^, at which the T-line intensity was still significantly higher than that of the negative control. Therefore, the visual limit of detection (vLOD) for TM using PDANs-based LFIA was determined to be 5 ng mL^−1^. To further evaluate the quantitative performance of the PDANs-based LFIA for TM detection, the RGB value of T-line was analyzed. A significant negative correlation was observed between the T-line RGB value and TM concentration, exhibiting a good linear relationship in the range of 5–100 ng mL^−1^ (*R*^2^ = 0.9879) (Fig. 4b). Meanwhile, an AuNPs-based LFIA was also constructed for TM detection. Under the optimized conditions, TM standard solutions were analyzed by AuNPs-based LFIA, and a vLOD of 10 ng mL^−1^ was obtained (Fig. S7). In comparison, the PDANs-based LFIA achieved a lower vLOD for TM detection.

For PIC detection, the analytical performance of the LFIA was evaluated in a similar manner. Standard PIC solutions in the concentration range of 1000–5 ng mL^−1^ were tested. As shown in Fig. 4e, the vLOD was determined to be 50 ng mL^−1^. Moreover, a good linear relationship between the T-line RGB value and PIC concentration was obtained in the range of 50–500 ng mL^−1^, indicating that the PDANs-based LFIA platform is suitable for the quantitative analysis of PIC (Fig. 4f).

Moreover, signal intensity was further amplified in the photothermal mode due to the outstanding photothermal performance of PDANs. Upon NIR irradiation (808 nm, 2 W cm^−2^, 1 min), the PDANs on the T-line converted light into heat, and the thermal images and temperature values were recorded using handheld infrared camera, supporting the portability of the proposed photothermal readout strategy (Fig. S8). The temperature of the T-line exhibited a positive correlation with the concentration of target. When the concentration of TM and PIC reached 0.5 ng mL^−1^ and 20 ng mL^−1^, respectively, the T-line temperatures showed significant differences compared with the negative sample (Fig. 4c and g). Therefore, the LODs of the PDANs-based LFIA in the photothermal mode were determined to be 0.5 ng mL^−1^ for TM and 20 ng mL^−1^ for PIC. Compared with the colorimetric mode, the LODs were improved by around 10-fold for TM and 2.5-fold for PIC. In addition to enhanced sensitivity, the photothermal mode also exhibited excellent quantitative performance. Good linear relationships were obtained between the Δ*T* of T-line and the concentrations of TM and PIC over the ranges of 0.5–50 ng mL^−1^ and 20–500 ng mL^−1^, respectively (Fig. 4d and h). These results indicate that the photothermal mode of the PDANs-based LFIA enables significantly improved detection sensitivity. Furthermore, a comparison with recently reported colorimetric–photothermal dual-mode LFIA systems is provided in Table S3. Compared with multicomponent photothermal labels, PDANs offer a facile and practical signal label because of their simple synthesis and intrinsic colorimetric–photothermal properties.

To further assess the analytical reliability of the PDANs-based LFIA platform, its specificity was subsequently evaluated by a series of disease-related biomarkers in human serum, including carcinoembryonic antigen (CEA), alpha-fetoprotein (AFP), cardiac troponin I (cTn I), TM and PIC. As shown in Fig. 4i and j, obvious dark bands with low RGB values were observed on the T-line only in the presence of the corresponding target, whereas negligible signals were detected for the non-target biomarkers. These results demonstrate the high specificity of the PDANs-based LFIA platform for TM and PIC detection.

### 3.5 Application in clinical serum samples

To evaluate the quantitative accuracy and precision of PDANs-based LFIA platform in complex biological environments, blank human serum samples and human serum samples spiked with TM (20, 50, and 80 ng mL^−1^) and PIC (80, 200, and 400 ng mL^−1^) at different concentrations were analyzed by this platform. As shown in Fig. S9, no visible T-line signal was observed for the blank serum samples both in the TM or PIC assay, indicating that the serum matrix did not generate obvious nonspecific colorimetric signals.

**Fig. 5:**
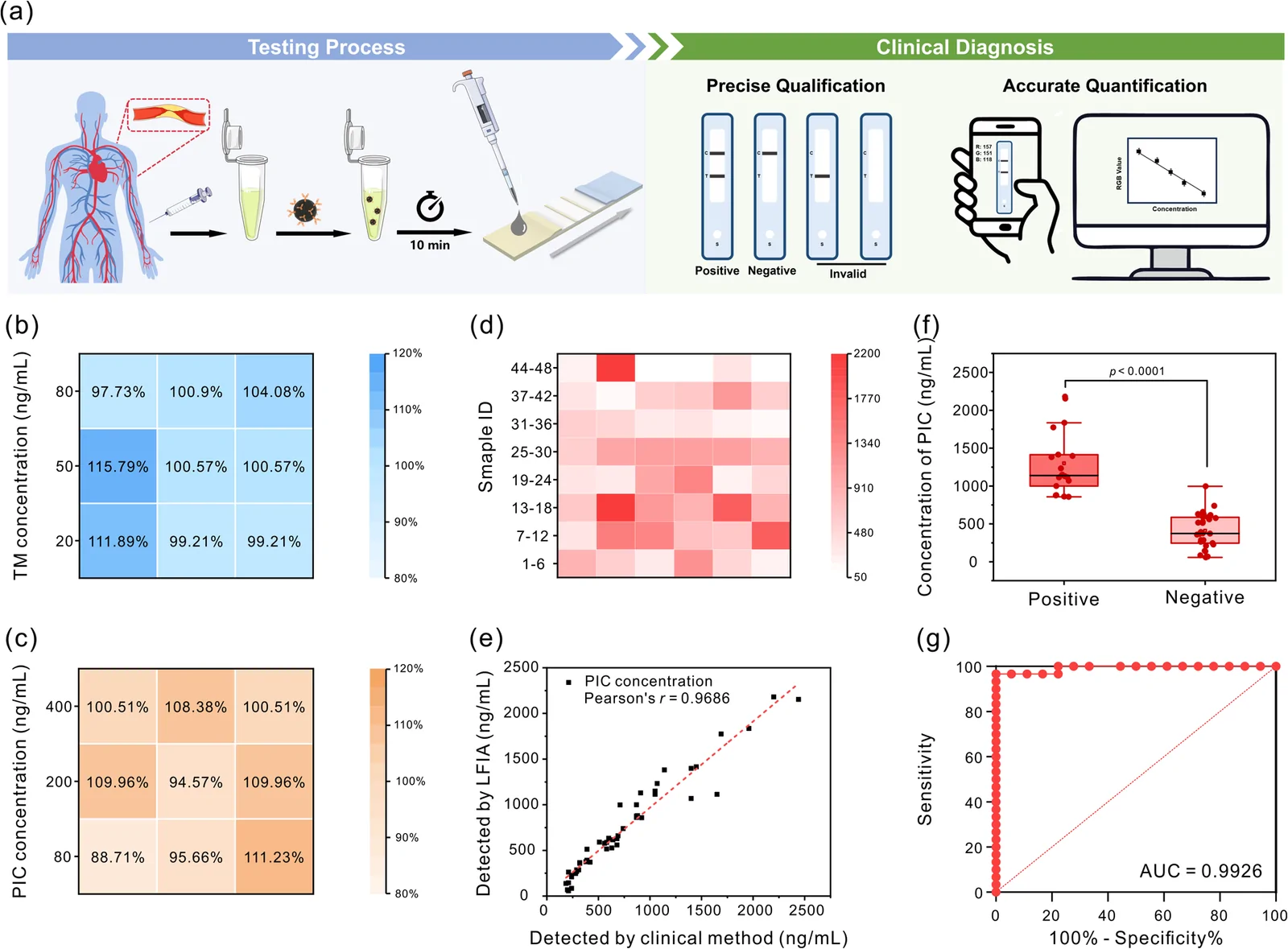
Clinical validation of the PDANs-based LFIA. (a) Schematic illustration of the testing procedure for TM and PIC in clinical serum samples. Recovery results of (b) TM and (c) PIC spiked into serum samples using the PDANs-based LFIA. (d) Heat map showing the detection results of PIC. (e) Correlation analysis between the PDANs-based LFIA and clinical method for quantifying the PIC concentrations. (f) Scatter plots of PIC concentrations detected by the PDANs-based LFIA for discriminating between negative and positive groups. Statistical significance was evaluated using a two-tailed unpaired *t*-test. (g) ROC curves of the PDANs-based LFIA for PIC detection.

For the spiked serum samples, the obtained RGB value fitted to the corresponding calibration curves to calculate the measured concentrations and recovery rates (Table S2). As shown in Fig. 5b and c, the recoveries of TM and PIC were in the ranges of 97.73–115.79% and 88.71–111.23%, respectively. These values are within the generally accepted recovery range for clinical analysis (80–120%), indicating the PDANs-based LFIA platform exhibits satisfactory quantitative accuracy and analytical precision for the detection of TM and PIC in complex biological matrices.

To further evaluate the potential applicability of the PDANs-based LFIA platform in real clinical samples, 48 human serum samples (18 positive and 30 negative samples) were analyzed (Fig. 5a). As shown in Fig. 5d, the detection results of these clinical samples are presented as a heatmap (the clinical reference value defined PIC concentrations ≥800 ng mL^−1^ as positive^40,41^). Meanwhile, the platform could efficiently discriminate between positive and negative samples, with a statistically significant difference (*p* < 0.0001), as shown in Fig. 5f. According to receiver operating characteristic (ROC) curve analysis, the area under the curve (AUC) reached 0.9926, with a 95% confidence interval of 0.9757–1.0000, indicating excellent diagnostic accuracy (Fig. 5g). A correlation analysis was further performed by plotting the PIC concentrations measured by the clinical method against those obtained by the PDANs-based LFIA (Fig. 5e). A good linear correlation was observed (*R*^2^ = 0.9686), indicating PDANs-based LFIA exhibited acceptable quantitative performance for PIC detection.

Overall, the consistent performance observed in clinical serum samples highlights the potential of the PDANs-based LFIA for reliable clinical analysis of thrombotic biomarkers. Nevertheless, we agree that the current sample size relatively limited for definitive diagnostic accuracy assessment, and further validation using a larger independent clinical sample will be required.

## 4 Conclusions

In summary, we developed a PDANs-based LFIA for the simultaneous rapid detection of TM and PIC. Uniform and stable PDANs were synthesized, exhibiting excellent photothermal performance and enabling efficiently antibody conjugated *via* covalent interactions. Based on these PDANs, a highly sensitive dual-mode colorimetric–photothermal LFIA was established. Under optimized conditions, the vLODs of TM and PIC were 5 ng mL^−1^ and 50 ng mL^−1^, respectively, while photothermal signal enhancement further improved the LODs to 0.5 ng mL^−1^ and 20 ng mL^−1^. The platform also demonstrated high specificity for the targets. Moreover, the PDANs-based LFIA exhibited promising clinical application. Analysis of spiked sample showed satisfactory recoveries. The platform effective discriminated between positive and negative samples, and the ROC curve analysis showed excellent agreement with clinical methods (AUC = 0.9926), indicating the quantitative accuracy and reliability of PDANs-based LFIA. Overall, this study provides a rapid, convenient, and highly sensitive detection strategy, offering a reliable tool for early identification and intervention of thrombotic events and laying a foundation for point-of-care screening and clinical applications.

## Author contributions

Jun Yin: conceptualization, data curation, formal analysis, investigation, methodology, writing – review and editing. Yu Qiu: conceptualization, data curation, formal analysis, investigation, methodology and writing – original draft. Yao Nie: conceptualization, investigation, formal analysis and methodology. Jiansheng Ju: resources, formal analysis and methodology. Yanmin Ju: conceptualization, funding acquisition, project administration, resources and supervision. Fei Wang: conceptualization, funding acquisition, project administration, resources and supervision.

## Conflicts of interest

The authors declare no conflict of interest.

## Supplementary Material

RA-016-D6RA04164G-s001

## Data Availability

The data supporting this article have been included as part of the supplementary information (SI). Supplementary information: experimental methods, calculation parameters, and supplementary figures and tables. See DOI: https://doi.org/10.1039/d6ra04164g.
