# Supplementary material for: Polydopamine nanoparticle-based colorimetric–photothermal dual-mode lateral flow immunoassay for thromboembolism diagnosis
Source: RSC Adv. 2026 Aug 12;16(40):47147–54. doi: 10.1039/d6ra04164g (PMC13463704; doi:10.1039/d6ra04164g)
Supplement: Supplementary file 1 [file RA-016-D6RA04164G-s001.pdf]

Supplementary Information (SI) for RSC Advances.
This journal is © The Royal Society of Chemistry 2026

Supporting Information

Polydopamine Nanoparticle-Based Colorimetric-Photothermal Dual-Mode Lateral Flow Immunoassay for Thromboembolism Diagnosis

Jun Yin[†, a], Yu Qiu[†, b], Yao Nie [b], Jiansheng Ju [a], Yanmin Ju [b, *] and Fei Wang [a, *]

[a] Taixing Hospital of Traditional Chinese Medicine, Jiangsu Taizhou, 225400, China;

[b] College of Pharmacy, China Pharmaceutical University, Jiangsu Nanjing, 211198, China.

[*] Correspondence: juyanmin@cpu.edu.cn (Y.-M.Ju); fly-feier-fly@163.com (F.Wang)

[†] These authors contribute equally

Table of Contents

1. Materials 2

2. Characterization Methods 2

3. Results 3

1. Materials

All the reagents involved in this research were commercially available and used without further purification unless otherwise noted. Solvents were either employed as purchased or purified through standard laboratory procedures. Dopamine hydrochloride ($C_8H_{11}NO_2\cdot HCl$), Bovine serum albumin (BSA) was obtained from Aladdin (Shanghai, China). thrombomodulin (TM) and α_2-plasmin inhibitor–plasmin complex (PIC) were purchased from Bioworld Technology, Inc. (Nanjing, China). Goat anti-mouse IgG was purchased from Jackson ImmunoResearch Inc. Nitrocellulose (NC) membranes were obtained from Sartorius Corporation. Polyesters sample pads, polyvinyl chloride (PVC) backboard, and absorbent pads were sourced from Hangzhou Autokun Technology.

2. Methods

2.1 Characterization

Ultraviolet-visible (UV-Vis) spectrum was obtained on a Shimadzu UV-1800 spectrometer. Dynamic light scattering (DLS, Zeta Sizer Nanoseries, Malvern Instruments, Ltd.) was employed to characterize the hydrodynamic radius and zeta potential changes of nanoparticles. SEM images were obtained using a Hitachi S-4800 scanning electron microscope.

2.2 "COLOR PICKER" software

To obtain RGB values for quantitative analysis, the strip images were imported into the "COLOR PICKER" app. The procedure included three steps: (1) importing the strip images into the app; (2) selecting fixed points on the T line of each strip; and (3)

extracting the corresponding RGB values. The obtained RGB values were used for subsequent quantitative analysis.

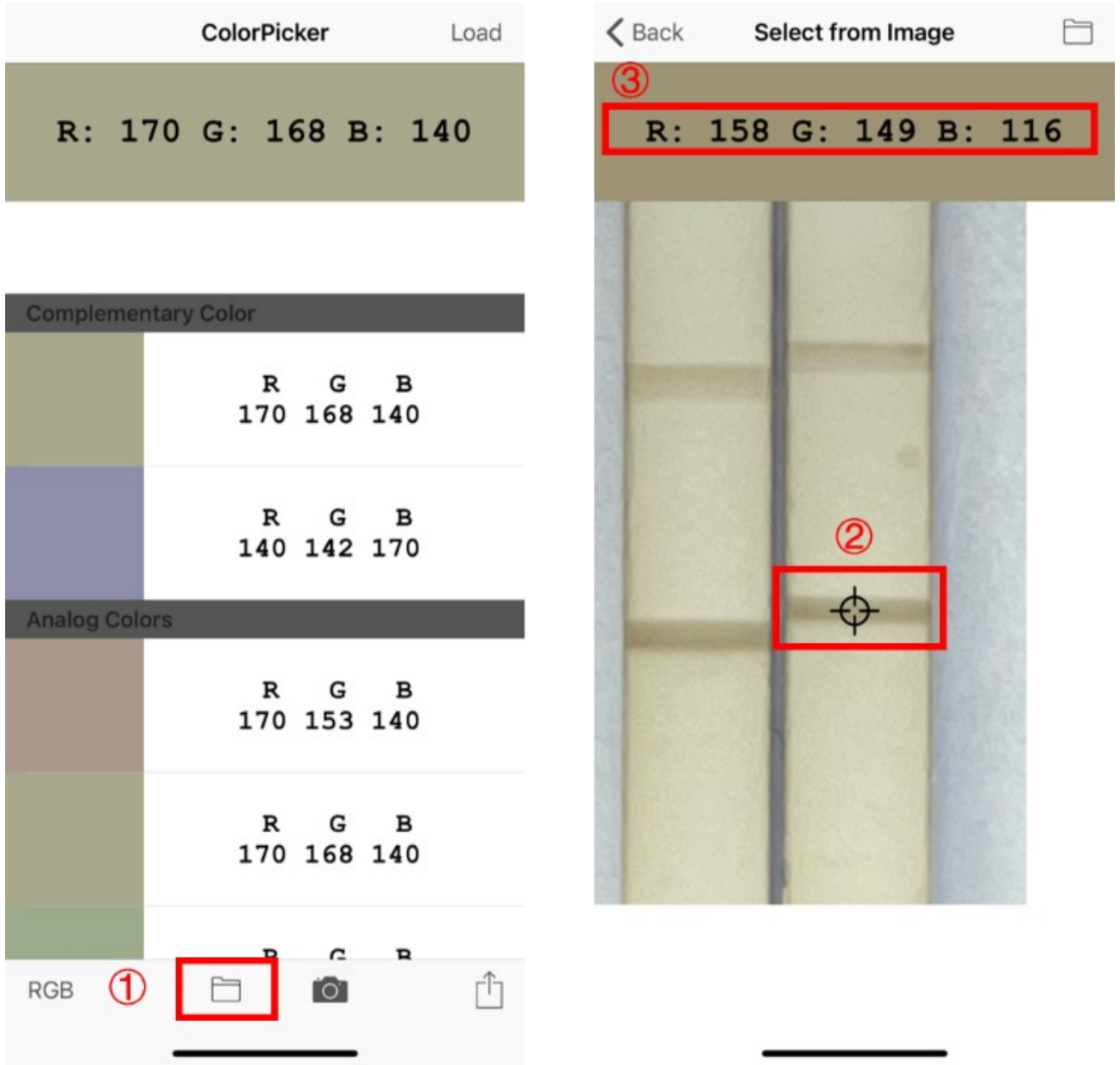

Figure S1. The steps to get the RGB value through Color Picker app.

2.3 Sample size estimation

The minimum required sample size was determined to be 44 participants per group using the t test function in G*Power 3.1.9.7 software, with assumptions of $\alpha = 0.05$, $\beta = 0.05$, and an effect size of 0.8.[1]

2.4 Photothermal conversion efficiency

The detailed calculation procedure and the actual values used for calculating the photothermal conversion efficiency are provided below.

Table S1. Parameters used for the calculation of photothermal conversion efficiency.

Parameter	Value
hS_{blank}	0.0033 W K^{-1}
hS	0.0025 W K^{-1}
T_{max}	83.6 °C
T_{surr}	26.7 °C
Q_{dis}	0.0066 W
I	2 W
A_{λ}	0.1108
η_T	30.10 %

3.Results

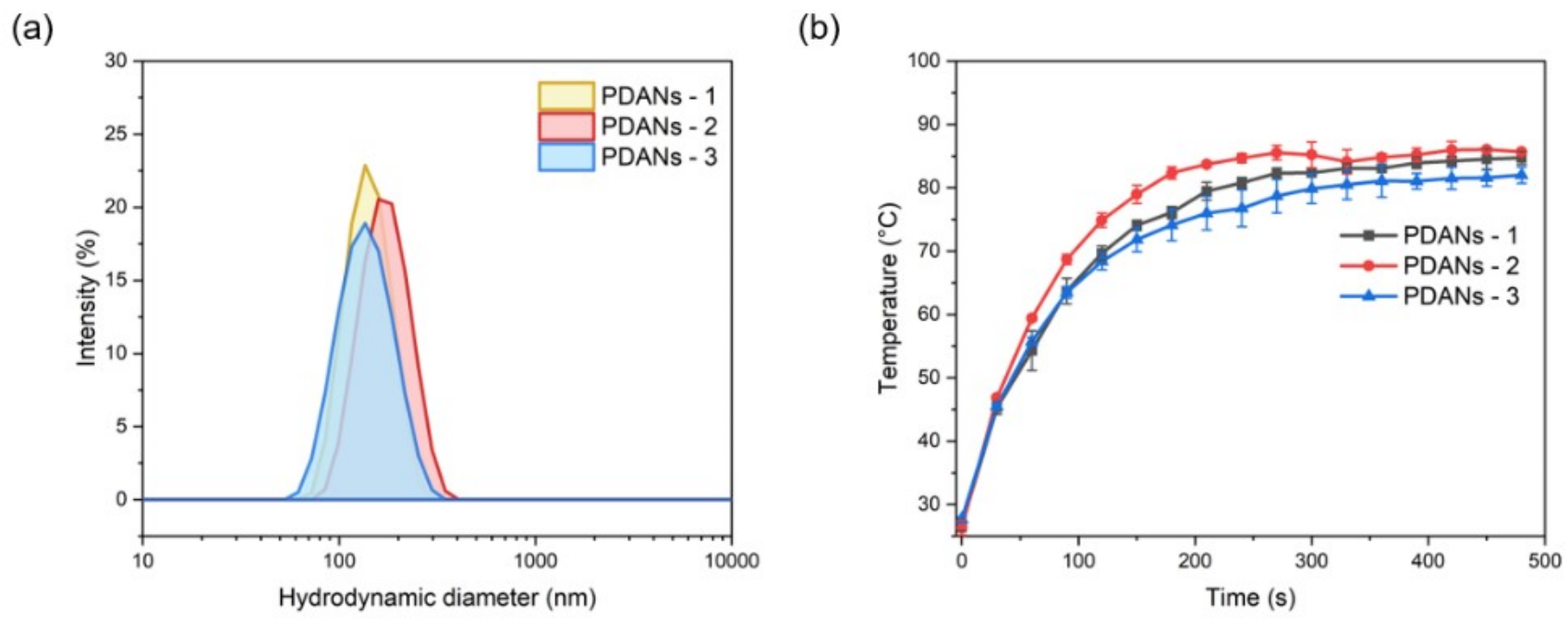

Figure S2. (a) Hydrodynamic size distributions, and (c) photothermal heating curves of three independently synthesized PDANs batches.

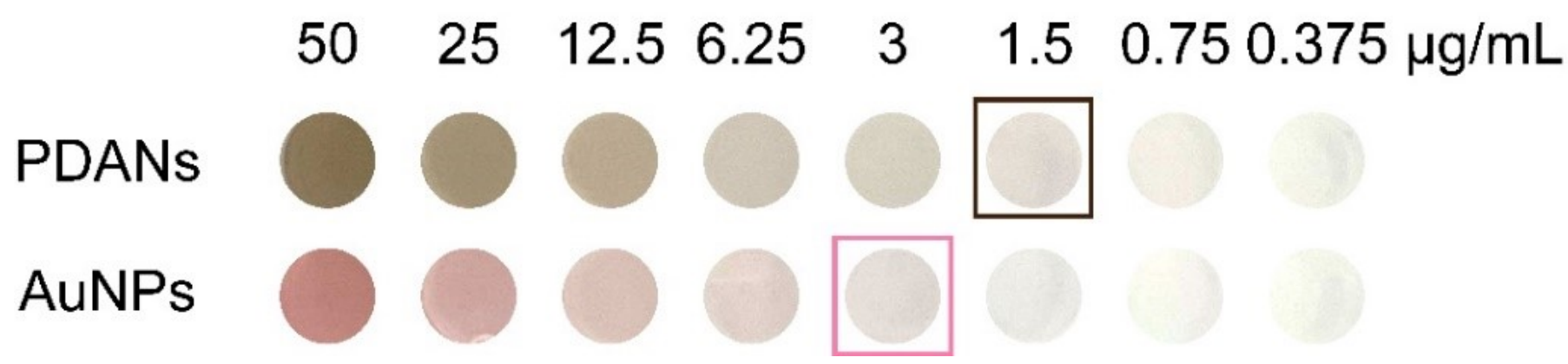

Figure S3. The typical photographs of different concentration PDANs and AuNPs.

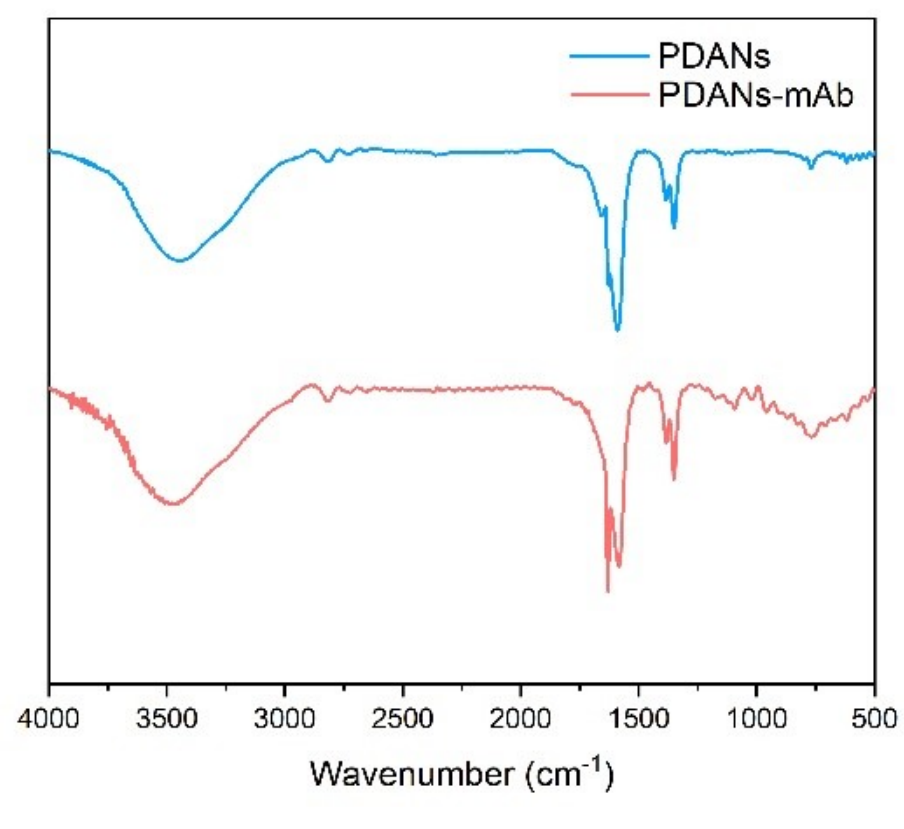

Figure S4. FTIR spectra of PDANs and PDANs - mAb.

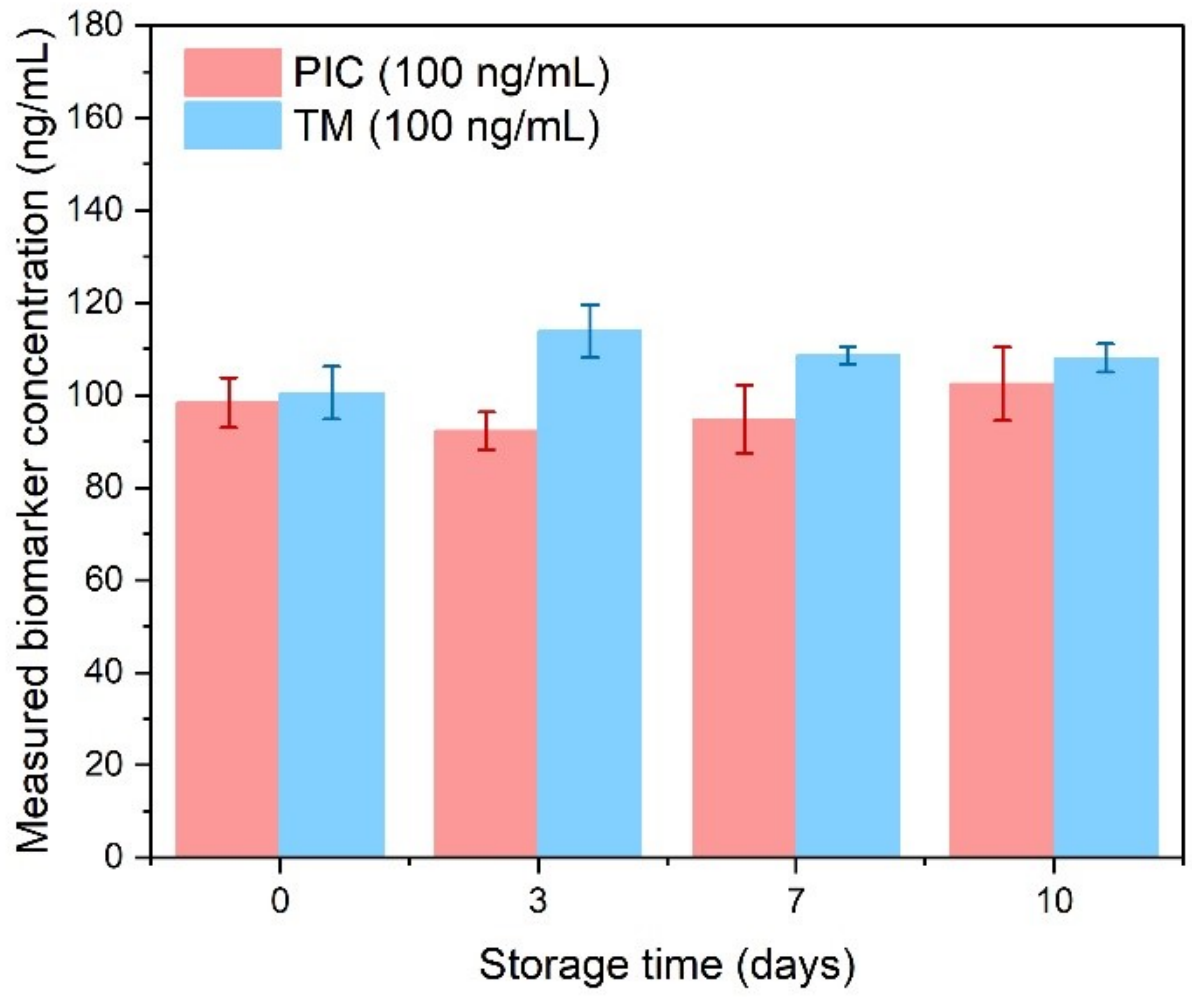

Figure S5. Storage stability of PDANs-antibody conjugates.

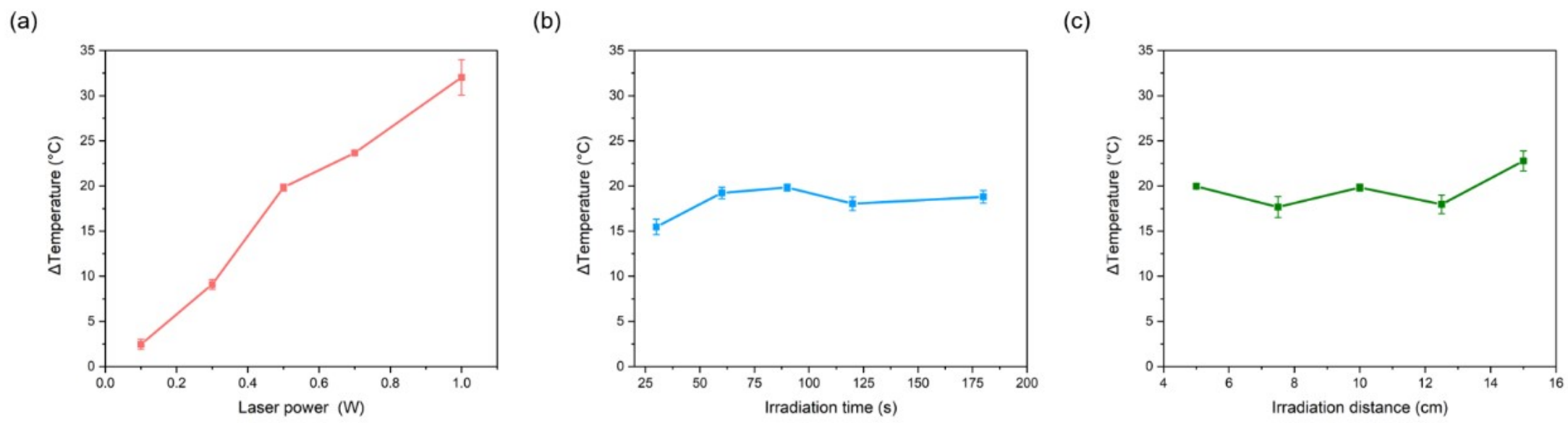

Figure S6. The effect of (a) 808 nm laser power, (b) irradiation time and (c) irradiation distance on ΔT.

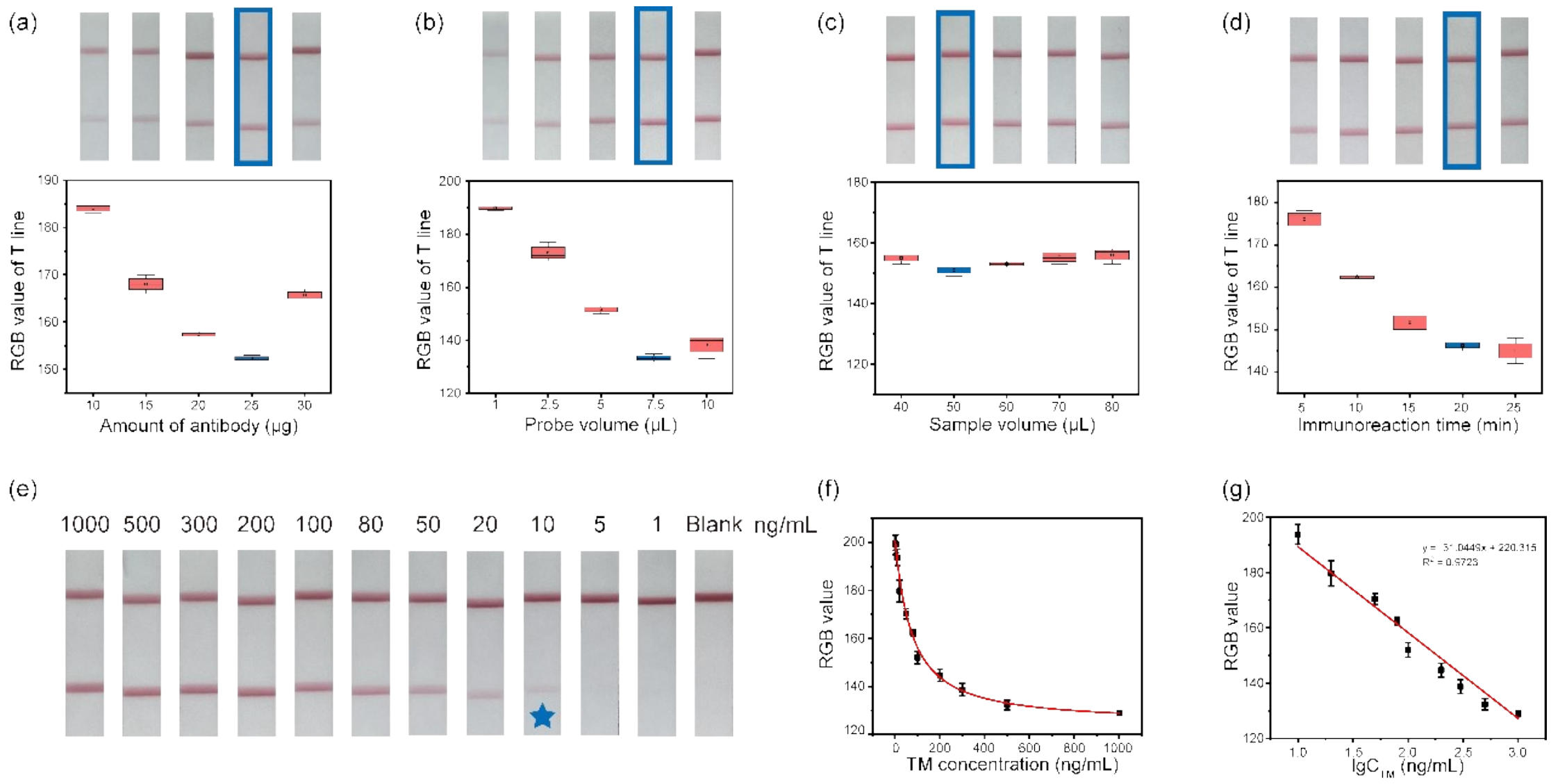

Figure S7. Optimization of experimental parameters and analytical performance of the AuNPs-based LFIA for TM detection. Detection results of TM under different conditions: (a) antibody amount, (b) probe volume, (c) sample volume, and (d) immunoreaction time between the sample and probe. (e–g) Colorimetric images and corresponding calibration curves of AuNPs-based LFIA for TM detection.

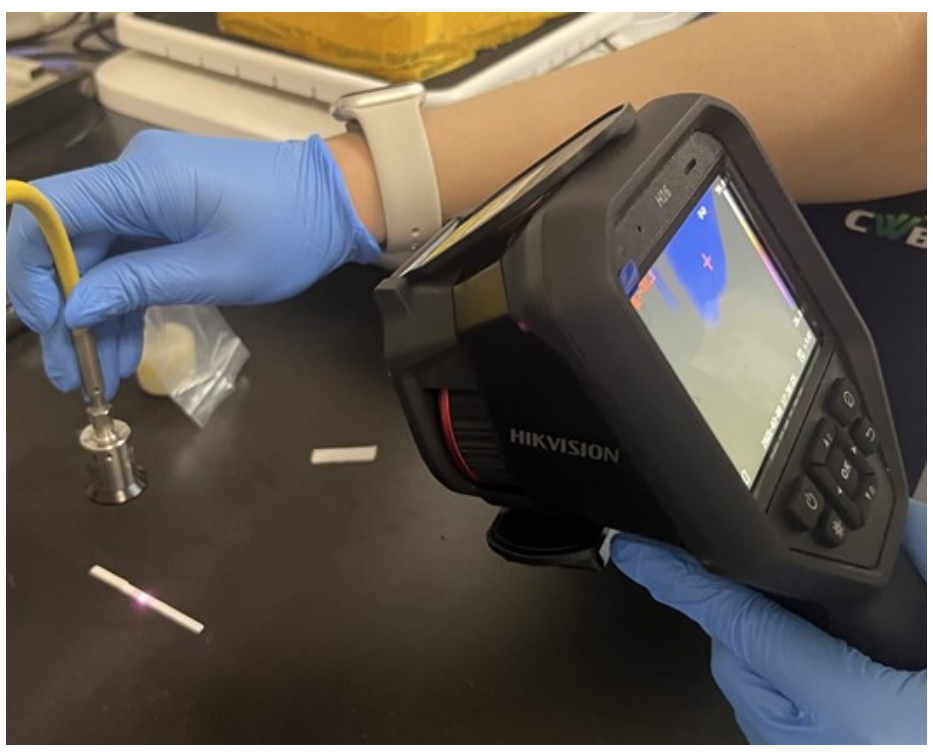

Figure S8. Photograph of the portable photothermal readout setup.

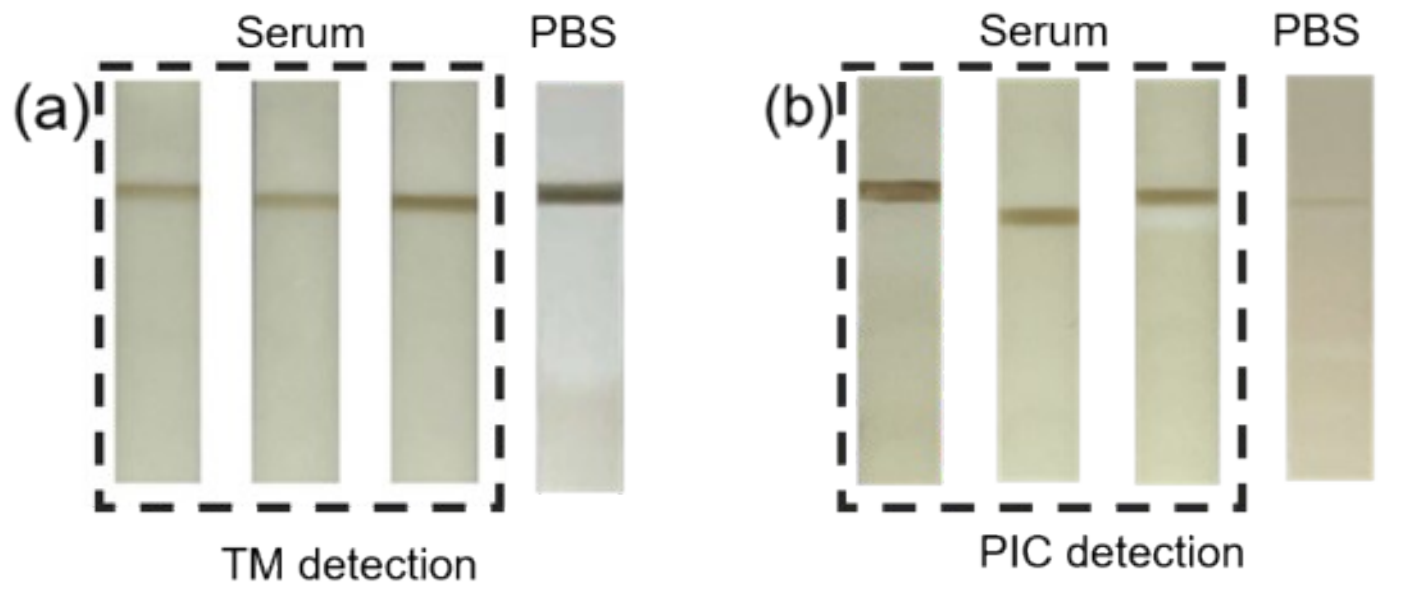

Figure S9 The images of blank serum samples tested using the PDANs-based LFIA.

Table S2. Recoveries of TM and PIC spiked in serum samples.

	Spiked concentration (ng/mL)	Mean ± SD (ng/mL)	Recovery (%)	CV(%)
TM	20	20.68 ± 1.46	103.43	7.08
	50	52.82 ± 4.39	105.64	8.32
	80	80.72 ± 2.54	100.90	3.14
PIC	80	78.83 ± 9.22	98.53	11.70
	200	209.66 ± 17.77	104.83	8.47
	400	412.52 ± 18.17	103.13	4.40

Table S3. Comparison of PDAN-based LFIA with recently reported colorimetric-photothermal dual-mode LFIA systems.

Materials	Detection Time (min)	Colorimetric		Photothermal		Detection	Reference
		LOD (ng/mL)	Line Range (ng/mL)	LOD (ng/mL)	Line Range (ng/mL)		
Au@PdPt NPs	10	0.059	0.11–1.00	0.038	0.07–0.72	Ofloxacin detection	2
Au core-petal NPs	25	1.68	2–64	0.58	1–256	Staphylococcal enterotoxin B	3
BP-Au NPs	13	0.25	0.01–100	0.0043	0.01–1000	Zearalenone	4
FY6@DSPE-PEG NPs	12	0.11	-	0.031	0.1–10	Aflatoxin B1 (AFB1)	5
Goldnanocages-4	30	1.8	75–2	0.00151	0.001–5	Influenza A (H1N1)	6
Platinum-confined gold nanocages	20	0.781	0.098–25	0.015	0.024–100	Chlorantraniliprole	7
Cu-anchored PDA	12	8.7	$10–10^{6}$	0.22	$1–10^{6}$	Aspergillus flavus	8
Au-Pd nanoprobes	12	0.263	0.125–1	0.142	0.125–1	Progesterone	9
Meso-UiO 66	10	49.26	$10^{2}–10^{4}$	24.59	$50–10^{4}$	Chikungunya virus	10
Plasmonic gold nanostars	35	1	0.60–18.43	0.28	0.10–27.49	Carbendazim	11
Au@Pt NPs	15	0.1	0.1–10	0.036	0.1–10	cTnI	12
PDANs	25	5	5-100	0.5	50-500	TM	This work
	10	50	0.5-50	20	20-500	PIC	

1. H. Kang, J Educ Eval Health Prof, 2021, 18, 17.
2. J. Zhang, L. Nan, L. Luo, Z. Li, S. Zheng, X. Xia, C. Xing, Z. Wang, Y. Pan and K. Wen, Mikrochim Acta, 2025, 192, 503.
3. H. Wu, Y. Li, Y. Li, Y. Cui, C. Jia, J. Wang, J. Pan, G. Yu, X. Zhang, X. Wang, P. Guo and Y. Ji, Chemical Engineering Journal, 2023, 470.
4. Y. Zhang, Z. Wang, X. Qu, J. Zhou, H. Yang, W. Wang and C. Yang, Sensors and Actuators B: Chemical, 2023, 390.
5. Q. Wang, J. Wang, Y. He and J. Wang, Food Chem, 2026, 505, 147911.
6. S. Atta, Y. Zhao, S. Sanchez, S. V. Yampolsky and T. Vo-Dinh, Anal Chem, 2025, 97, 6427-6437.
7. Z. Chen, Z. Zheng, Y. Zhang, J. Peng and W. Lai, Anal Chem, 2026, 98, 14436-14446.
8. M. Liang, X. Cai, Y. Gao, H. Yan, J. Fu, X. Tang, Q. Zhang and P. Li, Biosens Bioelectron, 2022, 213, 114435.
9. X. Guo, Z. Li, J. Guo, Z. Liang, S. Chang, Y. Xu, Y. Wang, J. Wang and Y. Ji, Food Chem, 2026, 506, 147916.
10. H. Chen, F. Zhong, Z. Li, Y. Di, J. Cao, C. Wang, B. Gu and L. Wang, Sensors and Actuators B: Chemical, 2026, 466.
11. L. Liu, T. Zhang, Q. Wu, L. Xie, Q. Zhao, Y. Zhang, Y. Cui, C. Wang and Y. He, Talanta, 2025, 281, 126891.
12. H. Tan, X. Cao, C. Xu, Y. Zhang, H. Li, H. Yan and M. Su, Mikrochim Acta, 2026, 193.
